# Transactions of the Medical Society of the State of New York

**Published:** 1871-03

**Authors:** 


### Transactions of the Medical Society of the State of New York.

We are indebted to some of our friends, probably Dr. William H. Bailey, the efficient and worthy *Secretary*, for re-print copies of the Transactions of the Medical Society of the State of New York, from 1807 to 1831, and from 1840 to 1843. These volumes are very valuable, enabling many physicians and libraries to complete their sets. As is well-known, they contain many of the most valuable papers presented to the profession during these periods, and are on this account valuable—doubly valuable as completing sets of the Transactions of our State Medical Society, "which has proved to be the prominent medical society of the country."—Dr. Bailey.

The prosperity and usefulness of the society, for the past years, has been greatly due to the efficiency and ability with which Dr. William H. Bailey has discharged the duties of the office of *Secretary*; and the medical profession of the state will not fail to recognize its obligations to him. The late Dr. H. D. Willard, former secretary, will also, in this connection, be remembered with gratitude.

